# Can robotic assistance mitigate the negative impact of sleep deprivation on surgical performance in microsurgical tasks?

**DOI:** 10.1016/j.jpra.2025.05.008

**Published:** 2025-05-30

**Authors:** Helena Frieberg, Olof Engström, Anna Nilsson, Villiam Vejbrink Kildal, Maria Mani

**Affiliations:** a Department of Surgery, Section of Plastic and Maxillofacial Surgery, Uppsala University, Uppsala, Sweden; b Department of Clinical Science and Education, Section of Anesthesiology and Intensive Care, Södersjukhuset, Karolinska Institutet, Stockholm, Sweden

**Keywords:** Lymphatic surgery, Microsurgery, Supramicrosurgery, Robotic surgery, Robot-assisted surgery, Surgical performance

## Abstract

**Introduction:**

Robotic surgery has expanded across various surgical disciplines, and although its application in microsurgery remains relatively novel, it has shown promise in a variety of microsurgical procedures. This study investigates how surgeons’ preoperative levels of sleep and stress influence perceived workload during manual and robot-assisted anastomoses in a laboratory setting.

**Methods:**

Seventeen participants with varying degrees of surgical experience performed a total of 149 anastomoses reporting pre-anastomosis sleep quality and stress levels as well as experienced workload factors during anastomosis such as perceived effort, frustration, and overall sense of performance.

**Results:**

Poor sleep quality increases the perceived exertion when performing manual anastomoses (*p* < 0.001) but not when performing anastomoses using robot assistance. The difference between the robot assisted group and the manual group was not seen when studying stress levels.

**Conclusion:**

Robot-assisted surgery may mitigate the negative impact of sleep deprivation on subjective workload during microsurgical anastomoses. The impact of sleep and stress on surgical performance, including anastomosis completion times and patency, requires further investigation.

## Introduction

Robotic surgery has been popularized since the turn of the century in many surgical disciplines, such as general surgery and urology and several different platforms are widely available.^1^ Although these have been used successfully in their respective fields, concerns have been raised about the instruments being too large and powerful to be optimal for microsurgical tasks,^2^ which has led to the development of dedicated microsurgical systems.^3^ An aspect that can be considered beneficial with robotic assistance in a microsurgical context is that the robot provides motion scaling and tremor filtration.^4^ Through motion scaling the surgeon’s motions are scaled down, allowing for more precise handling of tissues and instruments on a microscopic level, and tremor filtration provides for the removal of the natural tremor of the human hand, facilitating agility and precision, offering a new level of precision previously unattainable in manual surgery. Another potential benefit of robot-assisted surgery is the possibility of enhanced ergonomics since the adjustment of the control console can be tailored to the individual surgeon to reduce physical stress, which is particularly beneficial in long and demanding surgical procedures.^5^ Robot-assisted microsurgery has shown promise in allowing a broader range of surgeons to perform microsurgical anastomoses with greater precision^6^ and confidence, which could expand the availability of lymphatic surgery by improving the availability outside the most specialized centers, making it a compelling area of study.

In recent years, the advent of robotic assistance in microsurgery has emerged as a transformative development, particularly in the field of lymphatic surgery. Lymphatic microsurgery is by nature a discipline that requires great skill and dexterity due to the minute size of the structures being targeted. Compared to arteries and veins, the walls of lymphatic vessels are particularly thin and fragile, making the task of passing the needle through the vessel wall difficult.^7^ Through the progression of microsurgery to supermicrosurgery, targeting vessels smaller than 0,8 mm,^8^ we are getting closer to the limits of human performance, which is likely one of the reasons why interest in robot assistance has grown over the last few years.

Thus far robot assistance in lymphatic microsurgery has successfully been used for several different types of procedures, such as lymphovenous anastomosis (LVA), lympholymphatic anastomosis (LLA), and free vascular lymph node transfer,^2^^,^^9^, ^10^, ^11^, ^12^ and although the technique has shown great promise, concerns have been raised about the steep learning curve.^3^

Several aspects must be considered when evaluating the learning process of robot assistance in microsurgery, including task duration, anastomosis quality and the rate of improvement with training.^13^ Previous studies on skill acquisition in this field have shown the potential of developing proficiency regardless of previous microsurgical experience.^6^^,^^14^^,^^15^ While studies have primarily focused on anastomosis times, patency rates and frequency of errors,^16^^,^^17^ less is known about the subjective experience of using robot assistance for microsurgical reconstruction.

The aim of this study is to investigate how surgeons' self-reported levels of stress and sleep before anastomosis surgery – performed either by hand or with robot assistance—impacts workload scores during anastomosis surgery in a laboratory setting.

## Methods

The principles outlined in the Declaration of Helsinki have been followed. Approval was granted by the Ethics Committee of Uppsala University. The registration number of the ethical approval is DNr 2019–0504. Seventeen physicians from the Department of Plastic and Maxillofacial Surgery at Uppsala University Hospital participated. The physicians had varying degrees of surgical expertise and were grouped in two groups: short (no previous surgical experience and surgeons in training) and long (trained microsurgeons). Each participant completed between five and ten sessions, including one anastomosis by hand and one with robot assistance, using the MUSA-2 microsurgical robot (*MicroSure, Eindhoven, The Netherlands*), for each session. Before performing the first robot-assisted anastomosis, two training exercises were completed to gain familiarity with and to understand the MUSA-2 and its range of motion. Anastomoses were performed on 2 mm diameter synthetic polyvinyl alcohol vessels (*WetLab Corp*, Japan) using 9–0 Ethilon sutures.

Immediately before each session, each participant was asked to rate their sleep quality, motivation to perform the anastomosis, and stress level, see Table 1.

**Table 1:** Statements to assess stress, sleep and motivation.

Estimated quality of sleep in the last 24h	Good (3)	Moderate (2)	Poor (1)
Estimated stress level in the last 24h	Low (3)	Moderate (2)	High (1)

Text: Sleep quality and stress level were rated at each session from 1–3.

To evaluate the perceived exertion required, the participants also rated their experience of each anastomosis according to mental, physical, and temporal demand, effort required, perceived frustration, and overall perceived performance (see Table 2).

**Table 2:** Questions about perceived exertion during anastomosis.

Category	Question	Score
**Mental**	*How mentally demanding were the tasks in the training?*	*1 – 20*
**Physical**	*How physically demanding were the tasks in the training?*	*1 – 20*
**Temporal**	*How hurried or rushed were you during the training exercise?*	*1 – 20*
**Performance**	*How successful were you in accomplishing the training exercise?*	*1 – 20*
**Effort**	*How hard did you have to work to accomplish your level of performance?*	*1 – 20*
**Frustration**	*How insecure, discouraged, irritated, stressed, and annoyed were you?*	*1 – 20*

Text: To rate the perceived exertion required for each anastomosis, the participants rated their experience of each anastomosis according to mental, physical, and temporal demand, effort required, frustration perceived, and the overall perceived performance of each anastomosis on a scale of 1 (very low/perfect) to 20 (very high/failed).

Data were analyzed using linear mixed-effects models to assess the impact of several factors on six workload outcomes: mental, physical, temporal, performance, effort, and frustration scores. The models included sleep quality, stress level, experience, surgical method (robot-assisted anastomosis vs. manual anastomosis), and the number of anastomoses performed as fixed effects. Interaction terms between surgical methods and these covariates were included to explore differential impacts. Surgeon ID was incorporated as a random intercept to account for individual variability among surgeons. The models were reduced by removing non-significant terms to minimize the risk of overfitting. Post-hoc pairwise comparisons were conducted specifically for sleep quality and stress level within each surgical method, using estimated marginal means. Tukey adjustments for multiple comparisons were applied to control for family-wise error rates. This approach enabled the identification of specific effects of sleep quality and stress level on workload scores, while accounting for other influential factors such as experience and surgical method. A p-value of <0.05 was considered statistically significant.

## Results

Five participants were included in the group with long experience (microsurgeons) and twelve in the group with short experience (five surgeons in training and seven participants without any previous surgical training). A total of 46 and 103 anastomoses were made in the long experience group and short experience group, respectively. The total consists of both modalities (manual anastomoses and robot-assisted anastomoses).

**Figure 1:**
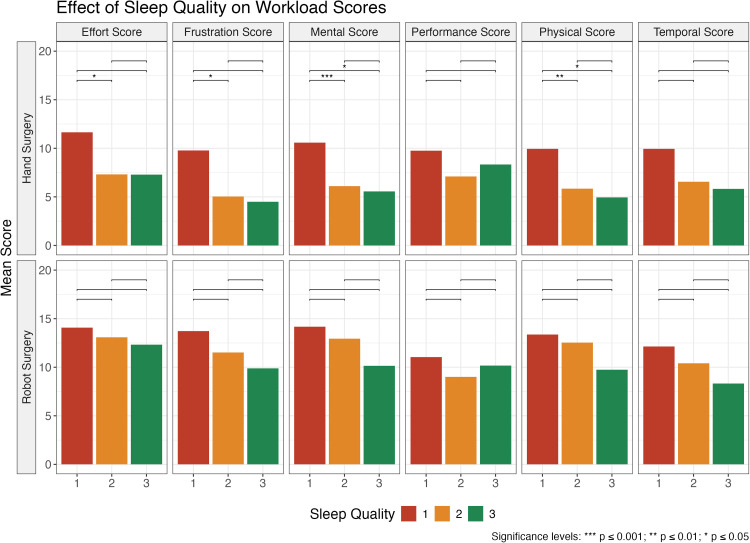
Plot showing how surgeons' self-reported sleep quality affects workload scores, with significance brackets indicating whether the differences between levels are statistically significant. Good sleep quality is represented by the green bar.

Better reported sleep quality was generally associated with lower mean workload scores, regardless of method, but always higher for robot-assisted anastomoses than for manual anastomoses (Figure 1). Poor sleep quality always led to the highest mean workload scores. Good sleep quality generally yielded lower mean workload scores, although the difference compared to moderate sleep quality was not as big as for poor sleep quality. For robot-assisted anastomoses, no significant differences in workload scores were found when comparing different sleep quality groups. For manual anastomoses, on the other hand, effort and frustration scores were higher in the poor sleep quality group than in the moderate sleep quality group (*p* < 0.05). Furthermore, mental and physical scores were also higher after poor sleep than after good sleep (*p* < 0.05) and moderate sleep quality (mental: *p* < 0.001, physical: *p* < 0.01). No significant differences were found in the workload scores comparing moderate and good sleep quality.

**Figure 2:**
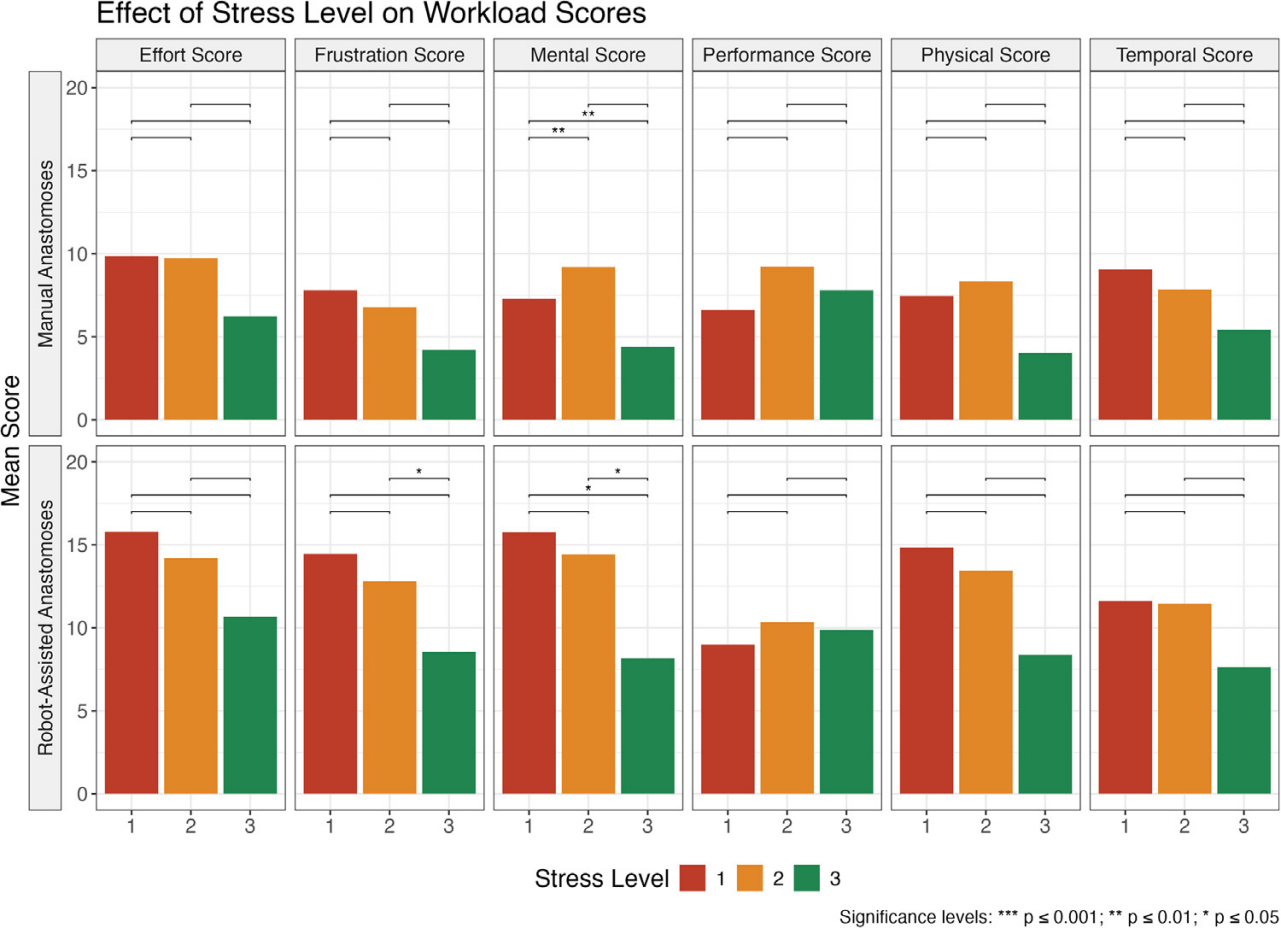
Plot showing how surgeons' self-reported stress levels affect workload scores, with significance brackets indicating whether the differences between levels are statistically significant. Low stress level is represented by the green bar.

Regardless of previous experience, lower reported stress levels were associated with lower mean workload scores regardless of technique, but all scores were higher for robot-assisted anastomoses than for manual anastomoses (Figure 2). However, high stress scores were not always associated with the highest mean workload scores. For mental, performance and physical scores in the manual anastomosis group, moderate stress level were associated with the highest mean workload scores. Low reported stress levels always resulted in the lowest mean workload scores in all groups. In the manual anastomosis group, the mental score was significantly lower in the low stress level group compared to the moderate (*p* < 0.001) and high (*p* < 0.001) stress level groups. In the robot-assisted group, the frustration score was higher in the moderate stress level group than in the low stress level group (*p* < 0.05), and mental score was also higher in the moderate stress level group than in the low stress level group (*p* < 0.05), and also in the high compared to the moderate stress level group (*p* < 0.05).

**Figure 3:**
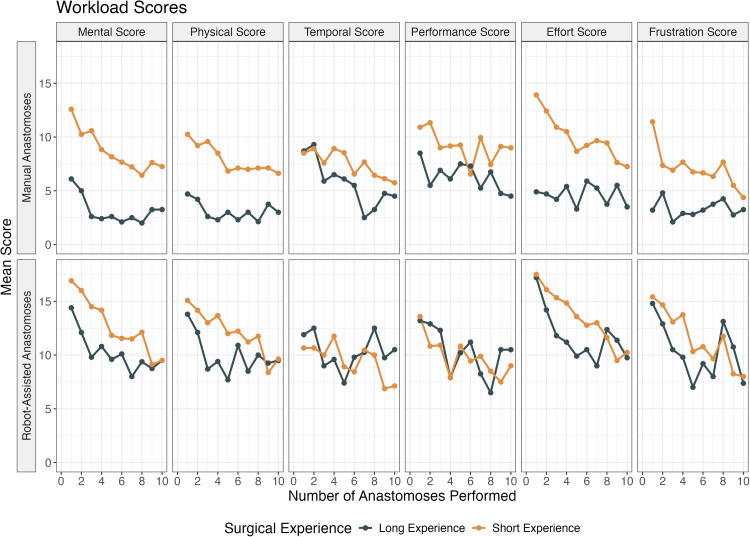
Plot showing mean workload scores for the different anastomosis methods (manual or robot-assisted), grouped by method and the surgeon’s length of experience. Short experience is defined as surgeons in training or physicians without previous surgical experience, while long experience is defined as trained microsurgeons.

With an increasing number of performed anastomoses, the mean workload scores showed a tendency to decrease regardless of group and technique (Figure 3). The decreasing trend was more pronounced for participants with short experience performing manual anastomoses. With robot assistance, the scores showed similarities between the groups. Temporal and performance scores did not show a steady decrease but showed more of a tendency to fluctuate over sessions. However, the mean workload scores all appeared to be lower for the tenth anastomosis compared to the first, regardless of previous experience and method.

## Discussion

The current study indicates that poor sleep quality affects the perceived exertion when performing manual anastomoses by hand but not when suturing with robot assistance. Specifically, poor sleep quality affected the mental and physical demands, effort, and frustration during manual anastomoses, but did not impact participants’ feelings of being stressed or rushed (temporal demand) or their perceived success of the task (performance). For robot assistance, the perceived sleep quality did not have any significant effect on workload scores. The workload scores for robot assistance were generally higher, which may indicate that using robot assistance is already demanding for those unfamiliar with the technique, potentially influencing scores regardless of sleep quality, as none of our participants had previous experience in robot assisted surgery.

In contrast, the perceived stress level affected workload scores for both robot-assisted and manual anastomoses. Higher stress levels were associated with higher mental demand, regardless of stress level and modality. The stress level also influenced reported frustration levels when using robot assistance but did not affect manual anastomoses. For the other workload scores, ie physical, temporal, performance, and effort, stress level did not appear to affect the average scores. Our results suggest that robot assistance could help mitigate the negative effects of poor sleep but not of high stress levels.

Our results, in general, showed lower scores for manual anastomosis than for robot-assisted anastomosis, where the differences between experienced and inexperienced also were greater. Four of our participants had previous experience in robot assisted laparocopic surgery, and one had used the Da Vinci robotic system, but none had any prior exposure to robot-assisted microsurgery. Despite this, the experienced microsurgeons scored lower when using robot assistance. Thus, our data suggest that manual anastomoses resulted in lower exertion, less frustration, and better mental and physical performance. Potentially, greater exertion scores can be explained by the limited exposure to robotic systems as all participants had experience of suturing single stitches manually beforehand. However, it also raises the question of whether current robotic systems may be as efficient as suggested theoretically. Future studies should investigate whether extended training with robot assistance mitigates these differences or if design improvements are necessary to optimize the efficiency and usability of the robot systems available today. Additionally, exploring how stress and sleep deprivation affect robot assisted performance could provide valuable insights into how these systems may be adapted to better support surgeons in high-pressure environments.

Results from previous studies have focused on whether sleep deprivation affects surgical performance, using outcomes such as errors, anastomosis completion time, anastomosis patency, complication rate, and surgical outcome.^18^^,^^19^ Ito et al. did not find any decline in performance under sleep deprivation,^19^ however, studies on internal medicine residents have shown inferior performance in attention, vigilance, and working memory tasks.^20^^,^^21^ Sleep deprivation has also been shown to affect dexterity^22^ and to lead to more errors in laparoscopic simulators,^23^ which has also been replicated in a microsurgical setting.^24^ Although the skill acquisition process in robot-assisted microsurgery has been studied^6^ less known about factors that may affect performance and the subjective experience of robot-assisted surgery. However, surgeons' subjective experience of exertion depending on sleep and stress is largely unknown.

Robot-assisted surgery has been widely implemented within many different surgical disciplines over the last couple of decades. Features such as motion scaling and tremor filtration have shown promise within plastic and reconstructive microsurgery, but the application of robot-assistance in microsurgery can still be considered to be in its early stages.^4^ In addition to increased precision, the utilization of robot-assistance can also provide improved ergonomics, which is essential during long procedures. These factors combined show promise in facilitating microsurgical tasks, which can hopefully enable surgeons with different backgrounds and degrees of previous experiences to perform robot-assisted microsurgical anastomoses earlier in their training, perhaps making the techniques more accessible in the future. As targeted anatomical structures become smaller the demand for precision increases, making robotic surgery particularly promising for lymphatic surgery, considering the delicate task of lymphatic anastomosis.^7^ Robot-assistance has been successfully used over a variety of applications within the field,^2^^,^^9^, ^10^, ^11^, ^12^ but there has been a concern about a steep learning curve.^3^

Limitations of this study include a relatively small sample size and the lack of validated workload scoring questionnaires. The subjective nature of stress level and sleep quality reporting may introduce potential bias, which could affect the accuracy of these measurements. There might also be a cognitive bias in the user’s experience. For example, individuals with a strong interest in technology might rate their robot-assisted performance more favorably, whereas surgeons with extensive manual experience mar evaluate it more critically due to the challenges associated with adopting a novel technique. This, however, was outside of the scope of this study. Furthermore, it should be noted that although the targeted structures are often smaller, the size of vessels (2 mm) and sutures (9–0) used in this study was chosen because of practical and financial reasons. It is reasonable to suggest that using smaller vessels and sutures, which more closely replicate the conditions of lymphatic surgery and supramicrosurgery, would enhance our findings, as smaller structures are generally associated with greater technical difficulty.

Whether robot assistance may improve results for surgeons working under stress or sleep deprivation due to long shifts and lengthy procedures warrants further investigation. Factors that could be explored include how sleep deprivation and stress could affect the quality of anastomosis, completion time, or skill acquisition. Whether the time of day would affect results could also be explored further,^24^ considering its implications on shift work.

## Conclusion

In the current study, poor sleep quality negatively impacted subjective workload scores for manual microsurgical anastomoses but not for robot-assisted anastomoses. Whether robot assistance can mitigate the negative effects of sleep deprivation requires further investigation.

## Ethics statement

The principles outlined in the Declaration of Helsinki have been followed. Approval was granted by the Regional Ethics Committee in Uppsala (2019–05,040).

## Funding

The study was funded by Lundberg’s grant foundation, received by Maria Mani in 2019.

## Declaration of competing interest

The Corresponding Author has been part of the Medical Advisory Board of Microsure since June 2022. All other authors have no financial disclosures or conflicts of interest.
